# Prevalence of Stress Urinary Incontinence in Elite Female Endurance Athletes

**DOI:** 10.2478/hukin-2014-0114

**Published:** 2014-12-30

**Authors:** Anna Poświata, Teresa Socha, Józef Opara

**Affiliations:** 1 Department of Physiotherapy, Jerzy Kukuczka Academy of Physical Education, Katowice, Poland.; 1 Department of Physical Education, Jerzy Kukuczka Academy of Physical Education, Katowice, Poland.

**Keywords:** stress urinary incontinence, female endurance athletes, prevalence

## Abstract

The goal of the study was to assess the prevalence of stress urinary incontinence in a group of elite female endurance athletes, as professional sport is one of the risk factors for stress urinary incontinence. SUI rates in the groups of female cross-country skiers and runners were compared to determine whether the training weather conditions like temperature and humidity influenced the prevalence of urinary incontinence. An anonymous questionnaire was distributed among 112 elite female athletes ie., 57 cross-country skiers and 55 runners. We used a short form of the Urogenital Distress Inventory (UDI-6) to assess the presence of SUI symptoms and the level of urogenital distress. Only women who had been practicing sport professionally for at least 3 years, on an international and national level, were included in the research. The study group consisted of 76% nulliparous and 24% parous women. 45.54% of all participants reported leakage of urine associated with sneezing or coughing which indicates stress urinary incontinence. 29.46% were not bothered by the urogenital distress symptoms. 42.86% of the participants were slightly bothered by the symptoms, 18.75% were moderately bothered, 8.04% were significantly bothered and 0.89% were heavily bothered. The absence of statistically significant differences between both groups seems to indicate that training weather conditions did not influence the prevalence of SUI in elite female endurance athletes.

## Introduction

Professional athletes often report different kinds of pathologies associated with the sports discipline they practise. Since female athletes have become important participants of professional sports, a wide range of sport-related pathologies needs to be taken into consideration, and, among them, urinary incontinence. Until now it has been believed that urinary incontinence is mainly a problem of the elderly and parous women. However, recent studies showed that young, physically fit, nulliparous women also suffer from UI. It seems that involvement in high-impact sports activity can increase the risk of urinary incontinence (Jean-Baptiste and Hermieu, 2010).

According to the International Continence Society, Urinary Incontinence is a storage symptom defined as involuntary loss of urine. Prevalence varies depending on study populations, investigation methods and the definition used. In a huge community-based epidemiological survey of female urinary incontinence carried out in Norway, 25% of all the respondents had some urinary leakage; the prevalence of UI depended on the age of the subjects. 50% of women who reported urinary leakage complained of stress incontinence, 11% had urge incontinence and 36% had mixed incontinence (Hannestad et al., 2000). In another cross-sectional study conducted in Turkey, 1012 women over the age of 18 years were interviewed using a questionnaire including “International Consultation on Incontinence Questionnaire - Short Form”. The overall prevalence of UI was 23.9% (n = 242). Among these women, 62 (25.6%) had urge, 80 (33.1%) stress and 100 (41.3%) mixed type of UI. The prevalence rate increased with advancing age (Kocak et al., 2005). Stress Urinary Incontinence is involuntary leakage on effort, exertion, sneezing or coughing resulting from changes in the fasciocutaneous tissues and pelvic floor muscles. These, in turn, can be a consequence of pregnancy and childbirth, pelvic organ prolapse, congenital anomalies within the genital tract, injuries and/or surgical interventions. Risk factors include hard physical work, perimenopausal estrogen deficiency, constitutional weakening of connective tissue, obesity, and, last but not least, professional sports. Greydanus et al. (2010) confirm that SUI occurs in about one-quarter of nulliparous young athletes at an average age of 20. Thus, the goal of the study was to determine the prevalence of stress urinary incontinence in a group of elite female endurance athletes since professional sport has been pointed out as a risk factor for SUI. On the other hand, there are two opposing hypotheses on how strenuous exercise or hard work might affect the pelvic floor: (1) physical activity may strengthen the pelvic floor muscles (PFM) and (2) physical activity may overload, stretch and weaken the pelvic floor (Bø, 2004). Our aim was also to compare the SUI rates in the groups of female cross-country skiers and runners to determine whether the training weather conditions like temperature and humidity might also influence the prevalence of urinary incontinence.

## Material and Methods

### Participants

112 elite Polish female athletes were invited to participate in the study. An anonymous questionnaire was distributed among 57 women practising cross-country skiing and 55 women who practised running. At the moment of data collection, the mean age of female runners was 29,49 ± 6,02 years and the mean age of women practising cross-country skiing was 26,61 ± 4,41 years old. The questionnaires were collected in the year 2012, mostly during competition. We included only those women who had been practising sports professionally for at least 3 years on an international or national level.

The exclusion criteria were age below 18 years, pregnancy and disorders that may affect bladder function, except, of course, urinary incontinence. The study group consisted of 76% nulliparous and 24% parous women. We enrolled 70% nulliparous and 30% parous cross-country skiers, 82% nulliparous and 18% parous runners. All studied female athletes practiced high-impact sports involving abrupt and repeated increases in intra-abdominal pressure that exceeded perineal floor resistance.

### Procedures

To assess the prevalence of SUI we used, among other questionnaires, a short form of the Urogenital Distress Inventory (UDI-6). This instrument assesses the symptoms of urinary incontinence (Uebersax et al., 1995). The UDI-6 questionnaire contains six questions regarding such symptoms as frequent urination, leakage related to feeling of urgency, leakage related to physical activity, coughing or sneezing, small amount of leakage (drops), difficulty empting bladder, pain or discomfort in lower abdominal or genital area. The 3 months preceding the study were taken into consideration, and the scale of 0 to 3 (0 - not at all, 1 - slightly, 2 - moderately, 3-greatly bothered) was applied. The average score was calculated and multiplied by 33 1/3 to put the score on the scale 0–100 (Uebersax et al., 1995). The scale was divided into four sections to closely describe the urogenital distress level: 1–25, 26–50, 51–75, 76–100. This helped to count the percentage of female athletes in each level. The questionnaire was conducted in both experimental groups, afterwards, the results were compared. We used the UDI-6 version validated and translated to the Polish language. The study protocol was approved by the Academy of Physical Education in Katowice (Poland) Ethics Committee, and conformed to the standards set by the Declaration of Helsinki.

### Statistical Analysis

The percentages were calculated to assess the prevalence of urinary incontinence symptoms in both study groups. The *chi*-square statistics were calculated to compare the distribution of symptoms and urogenital distress between the groups of female athletes practising cross-country skiing and running. The level of significance was set at p<0.05.

## Results

We found that 50.00% of all study subjects were losing small amounts of urine. In 27.68% of the respondents, incontinence was associated with a sense of urgency. 45.54% of the participants reported leakage of urine associated with sneezing or coughing, which indicated stress urinary incontinence symptoms. All together in the group reporting SUI symptoms and in that one which reported urge urinary incontinence, there were 18.75% female athletes, who reported mixed incontinence. Other problems associated with the urinary tract, which were pointed out in the questionnaire, were frequent urination (58.04%), pain or discomfort in lower abdominal or genital area (36.61%) and problems with bladder emptying (33.04%). There were no statistically significant differences between both groups.

**Figure 2:**
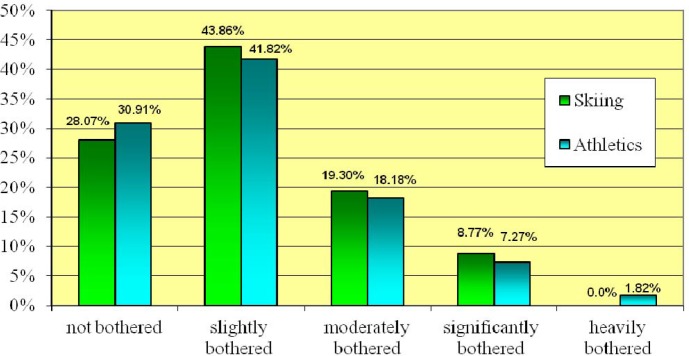
The level of Urogenital Distress

The obtained data also showed, on the scale from 0–100, the degree to which the respondents found the UI symptoms bothersome. 29.46% were not bothered by the urogenital distress symptoms. 42.86% of the participants were slightly bothered by the symptoms (1–25), 18.75% were moderately bothered (26–50), 8.04% were significantly bothered (51–75) and 0.89% were heavily bothered (76–100). Figure 2 shows the percentage in each group.

The analysis of the results indicates that elite female endurance athletes do suffer from urinary incontinence. No statistically significant differences were noted between the groups of professional female runners or cross-country skiers which indicates that training weather conditions do not influence the prevalence of urinary incontinence.

## Discussion

As outlined in the introduction, previous our study showed that a significant number of elite female athletes had the symptoms of stress urinary incontinence. Bø (2004) reported that because of sudden intra-abdominal pressure increases, pelvic floor muscles needed to be much stronger in elite athletes than in non athlete controls, while Silva et al. (2013) suggested that perineal pressure was decreased in female athletes compared to non athlete women. A lower perineal pressure correlates with increased symptoms of urinary incontinence and pelvic floor dysfunction. In a retrospective cohort study of female Olympians, Nygaard (1997) found that 35% of Olympic track and field participants had urinary leakage while competing at the Olympic Games. In general population, SUI has an observed prevalence of between 4% and 35% (Luber, 2004). Bø and Sundgot-Borgen (2010) concluded that female elite athletes had a high prevalence of stress and urge incontinence, which is also our conclusion. These researchers compared female elite athletes, aged 15 to 39 years, to a control group; 4% of the athletes and 33% of the controls were parous. It was found that the overall SUI prevalence was 41% in athletes and 39% in age-matched controls. On the other hand this difference was not statistically significant. In 2010, the same authors investigated 331 former elite athletes and 640 controls using a postal questionnaire including validated questions on UI. While competing in sport, 10.9% of the former elite athletes reported stress urinary incontinence. The prevalence of SUI in former elite athletes classified as participating in low, medium and high-impact activities was 5.3%, 10.7% and 13.0%, respectively. This was however a retrospective study, what might account for the difference between the results obtained in their and our investigations. Caylet (2006) assessed the prevalence of stress urinary incontinence and urge urinary incontinence in elite women athletes versus the general female population. An anonymous self-questionnaire was distributed among women aged 18 to 35 years. The first group comprised elite female athletes and the other non athlete women in the same age range. A total of 157 answers from elite athletes and 426 from control subjects were collected. The prevalence of urinary incontinence was 28% for athletes and 9.8% for the control subjects. Although there was no significant difference in the relative prevalence of SUI between the athletes and control subjects, the author concluded that the prevalence of urinary incontinence in women athletes was very high. The study of Carls (2007) also demonstrated that young female athletes participating in high-impact sports might be at a higher risk for urinary incontinence; over 25% of those completing the survey experienced incontinence. Simeone et al. (2010) collected an anonymous self-questionnaire from 623 female athletes aged 18 to 56 years, who were involved in 12 different sports; the prevalence of urinary incontinence was 30%. High-impact sports were frequently associated with incontinence, while low-impact sports with lower urinary tract symptoms. Stress incontinence was more frequent in hockey and volleyball players. Nygaard et al. (1990) considered 290 women regularly practicing recreational sport. In their study, 47% of participants had some degree of incontinence and noted that exercises involving repetitive bouncing were associated with the highest incidence of incontinence. These results are similar to ours, however, Nygaard et al. (1990) did not focus on professional sport and their rate of nulliparous women was only 22% compared to 76% in our study. They also showed that women, who practiced recreational sports, tended to change disciplines to those which did not involve so much jumping, eg., walking or swimming. Elite athletes cannot change the way they practice, even when symptoms of SUI start to appear. The study of Salvatore et al. (2009) also focused on recreational sports. The percentage of nulliparous women (ie. 75.1%) was similar to our group. Only 101 women (14.9%) of 679 study participants reported urinary incontinence, which might mean that recreational sport did not have such a significant impact on urinary incontinence as professional sport. Thyssen (2002) studied a group of 291 elite athletes and dancers, where 151 women (51.9%) had experienced urine loss. Urinary incontinence proportion in different physical activity areas was as follows: gymnastics: 56%, ballet: 43%, aerobics: 40%, badminton: 31%, volleyball: 30%, athletics: 25%, handball: 21% and basketball: 17%. Of those who leaked during sport, 95.2% experienced urine loss while training versus only 51.2% during competition. The activity most likely to provoke leakage was jumping. Fozzatti et al. (2012) evaluated the prevalence of stress urinary incontinence in 488 young nulliparous woman. The average age was 25.68 in the group of women who attended gyms and performed high-impact exercises and 24.45 in the control group, who did not attend gyms. Three questionnaires were used for the evaluation of stress urinary incontinence. There was a significant difference between groups. Women who attended gyms and performed high-impact exercises had a higher prevalence of urinary incontinence symptoms than those who did not take part in any high-impact exercise.

## Conclusions

Our study, based on the questionnaire results, confirms high prevalence of stress incontinence in female elite athletes practising high-impact sports. We can also conclude that weather conditions like temperature and humidity do not have an impact on the SUI symptoms, since we did not note statistically significant differences between both study groups.

The limitation of the study is the fact, that only the questionnaire was used to assess the prevalence of SUI symptoms. The UDI-6 questionnaire is known to be a solid tool, both for the clinical research and clinical practice. It correlates very well with pad tests, urodynamical parameters or with voiding diaries. Nevertheless, there are always some limitations regarding the reliability of the survey and its understanding by the participants. In future, more knowledge regarding urinary incontinence in general and the symptoms of SUI should be given to athletes, before the survey is handed out. Urodynamic tests or other urogenital diagnostic could be performed to receive more detailed information or more precise diagnosis. Further clinical studies should be performed, including diagnostic procedures, to analyze the prevalence of urinary incontinence among female athletes.

## Figures and Tables

**Figure 1:**
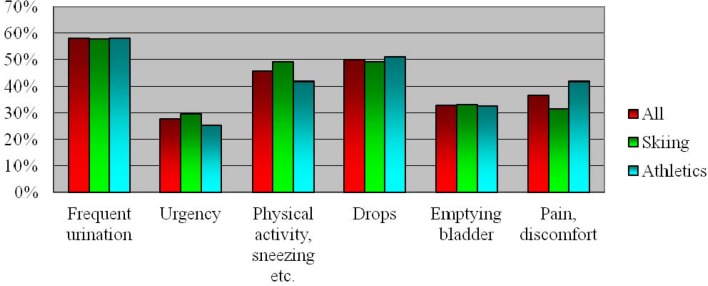
Prevalence of UI symptoms according to UDI-6
